# Human Cardiomyocyte Progenitor Cells in Co-culture with Rat Cardiomyocytes Form a Pro-arrhythmic Substrate: Evidence for Two Different Arrhythmogenic Mechanisms

**DOI:** 10.3389/fphys.2017.00797

**Published:** 2017-10-12

**Authors:** Nicoline W. Smit, Lucia Cócera Ortega, Anna M. D. Végh, Veronique M. F. Meijborg, Anke M. Smits, Mischa Klerk, Anke J. M. Tijsen, Hanno L. Tan, Marie-José H. T. Goumans, Gerard J. J. Boink, Ruben Coronel

**Affiliations:** ^1^Department of Clinical and Experimental Cardiology, Heart Center, Academic Medical Center, University of Amsterdam, Amsterdam, Netherlands; ^2^Netherlands Heart Institute, Utrecht, Netherlands; ^3^Department of Molecular Cell Biology, Leiden University Medical Center, Leiden, Netherlands; ^4^IHU Liryc, Electrophysiology and Heart Modeling Institute, Fondation Bordeaux Université, Bordeaux, France

**Keywords:** cardiomyocytes, progenitor cells, electrical mapping, arrhythmias (mechanisms), paracrine

## Abstract

**Background:** Cardiomyocyte progenitor cells (CMPCs) are a promising cell source for regenerative cell therapy to improve cardiac function after myocardial infarction. However, it is unknown whether undifferentiated CMPCs have arrhythmogenic risks. We investigate whether undifferentiated, regionally applied, human fetal CMPCs form a pro-arrhythmic substrate in co-culture with neonatal rat ventricular myocytes (NRVMs).

**Method:** Unipolar extracellular electrograms, derived from micro-electrode arrays (8 × 8 electrodes) containing monolayers of NRVMs (control), or co-cultures of NRVMs and locally seeded CMPCs were used to determine conduction velocity and the incidence of tachy-arrhythmias. Micro-electrodes were used to record action potentials. Conditioned medium (Cme) of CMPCs was used to distinguish between coupling or paracrine effects.

**Results:** Co-cultures demonstrated conduction slowing (5.6 ± 0.3 cm/s, *n* = 50) compared to control monolayers (13.4 ± 0.4 cm/s, *n* = 26) and monolayers subjected to Cme (13.7 ± 0.6 cm/s, *n* = 11, all *p* < 0.001). Furthermore, co-cultures had a more depolarized resting membrane than control monolayers (−47.3 ± 17.4 vs. −64.8 ± 7.7 mV, *p* < 0.001) and monolayers subjected to Cme (−64.4 ± 8.1 mV, *p* < 0.001). Upstroke velocity was significantly decreased in co-cultures and action potential duration was prolonged. The CMPC region was characterized by local ST-elevation in the recorded electrograms. The spontaneous rhythm was faster and tachy-arrhythmias occurred more often in co-cultured monolayers than in control monolayers (42.0 vs. 5.4%, *p* < 0.001).

**Conclusion:** CMPCs form a pro-arrhythmic substrate when co-cultured with neonatal cardiomyocytes. Electrical coupling between both cell types leads to current flow between a, slowly conducting, depolarized and the normal region leading to local ST-elevations and the occurrence of tachy-arrhythmias originating from the non-depolarized zone.

## Introduction

Regenerative cell therapies are being developed for patients suffering from heart failure and acute or chronic myocardial infarction (Laflamme and Murry, 2005). Ideally, the applied cells should replace the scar tissue by viable myocardial tissue and improve cardiac output accordingly. Up to date, there is no stem cell type, differentiated or undifferentiated, that is preferred and improvements in hemodynamic function are either limited (Meyer et al., 2006; Valina et al., 2007) or not seen at all (Hirsch et al., 2011; Jansen of Lorkeers et al., 2015). Secondly, *in vitro* (Chang et al., 2006; Askar et al., 2013; Ten Sande et al., 2016), and *in vivo* (Chong et al., 2014; Shiba et al., 2016) studies have shown that there is an inherent risk for the formation of a pro-arrhythmic substrate or the occurrence of arrhythmias after application (Menasche et al., 2008; Makkar et al., 2012).

The resting membrane potential (RMP) of a cardiomyocyte determines the availability of the voltage dependent sodium channels for activation. Once these ion channels are activated they initiate an action potential (AP). Cardiomyocytes have a more negative RMP (~-90 mV) than undifferentiated stem cells (e.g., mesenchymal stem cells; ~-35 mV) (Heubach et al., 2004). If coupling between stem cells and cardiomyocytes occurs, the electrotonic interaction may cause cardiomyocytes to become depolarized (Askar et al., 2013; Ten Sande et al., 2016) and less excitable. If this occurs in a spatially heterogeneous manner (e.g., cells applied via injections), this causes regional heterogeneous conduction slowing that can lead to unidirectional block and re-entrant arrhythmias (Lammers et al., 1990). We have previously shown that human adipose-derived stromal cells interact with cardiomyocytes through a direct coupling effect (by the formation of gap junctional channels between the cells) and induce heterogeneous conduction slowing (Ten Sande et al., 2016).

An alternative mechanism through which cells can form a pro-arrhythmic substrate is anatomical block. This is most apparent in studies where transplanted cells are unable to couple with cardiomyocytes (Leobon et al., 2003). Thirdly, it has been suggested that paracrine factors secreted by stem cells can alter the electrophysiology of cardiomyocytes by acting on the membrane potential (Pedrotty et al., 2009; Ten Sande et al., 2016), and/or the repolarization phase (Askar et al., 2013).

It is unclear whether the type of stem cell used determines the arrhythmogenic potential. Cardiac stem cells (CSCs) express (i) the stem cell factor receptor (c-Kit) (Beltrami et al., 2003), (ii) stem cell antigen-1 (Sca-1^+^) (Oh et al., 2003), or (iii) the homeodomain transcription factor (islet-1) (Laugwitz et al., 2005) and are capable of growing into cardiospheres (Messina et al., 2004). CSCs appear to be a competent source for cell-based therapies because of their intrinsic ability to differentiate toward cardiac lineages both *in vitro* (Goumans et al., 2007) and *in vivo* (Smits et al., 2009a). However, it has been suggested that the regenerative potential of autologous cells of elderly patients can be diminished due to age and risk factors present in the elderly population (Golpanian et al., 2015). Young (fetal cells) might, therefore, be more beneficial to use. The arrhythmogenic risks of undifferentiated fetal CSCs are unknown. Human (fetal) cardiac stem cells with progenitor cell properties (CMPCs) can be isolated with help of Sca-1- antibodies (Goumans et al., 2007).

Using an established *in vitro* model resembling cardiac tissue we demonstrate here that locally applied undifferentiated, human fetal CMPCs, form a pro-arrhythmic substrate. Our data indicate that CMPCs create a depolarized region through coupling to neonatal rat ventricular myocytes (NRVMs). This is evident from the action potentials (APs) measured with micro-electrodes, the presence of conduction slowing and the changes in ST-segment.

## Methods

All animal experiments were approved by the local Animal Experiments Committee (Academic Medical Center, University of Amsterdam) and carried out in accordance with national and institutional guidelines. Approval by the Medical Ethics committee of the Leiden University Medical Center and informed consent, in written form, was obtained to isolate CMPCs from human fetal hearts (17–20 weeks, elective abortion). All procedures were carried out in accordance with the principles set forth in the Declaration of Helsinki.

### Isolation and culturing of neonatal rat ventricular myocytes (NRVMs)

Isolation of NRVMs was done as previously described (Ten Sande et al., 2016). Briefly, hearts were rapidly explanted from 1- to- 2-day- old Wistar rats. The ventricles were cut into pieces and dissociated with trypsin (1 mg/mL; Becton Dickinson BV, Breda, The Netherlands) and collagenase type 2 (1 mg/mL, Worthington Vollenhove, The Netherlands, 230 units/mg). A pre-plating step was performed to minimize fibroblast contamination. NRVMs were plated on multi-electrode-arrays (MEAs, Multi-Channel Systems MCS GmbH, Reutlingen, Germany) coated with fibronectin (125 μg/ml BD Biosciences, Breda, The Netherlands) at a density of 1.4 × 10^5^ cells per cm^2^. MEAs consisted of an 8 × 8 electrode matrix, electrode diameter was 100 μm and interelectrode distance of 700 μm. NRVMs were cultured at 37°C and 5% CO_2_ in culture medium (M199 medium; Gibco) supplemented with 10% heat inactivated fetal bovine serum (FBS; Gibco), 1% HEPES (Gibco #5630-0-80), 5000 U/L penicillin-G (Sigma-Aldrich,#P7794), 2 mg/L vitamin B12 (Sigma-Aldrich, #V2876), 3.5 g/L glucose, 1% non-essential amino acids (Gibco, #11140-050), and 1% L-glutamine (Gibco,#25030-081), which was switched to 2% FBS at day three of culture.

### Isolation and culturing of human cardiomyocyte progenitor cells (CMPCs)

CMPCs were isolated from human fetal hearts obtained after elective abortion, as previously described (Smits et al., 2009b). In short, hearts were cut into small pieces and washed with M-buffer (PBS supplemented with 2 mM EDTA (Sigma-Aldrich, #E4884) and 1% FBS). After digestion with collagenase-A (500 ng/mL, Roche #10103578001), cells were passed through a cell strainer (40 μm) and incubated with anti-Sca-1-FITC antibody (Miltenyi Biotec, Anti-Sca-1 MicroBead kit (FITC, #130-092-529), followed by an incubation with anti-FITC microbeads. Magnetic activated cells sorting (MACS) using a MiniMACS separation column (Miltenyi Biotec, type MS^+^, #130-042-201) was used to separate cells that had bonded to the microbeads from the negative fraction. The fraction bound to the microbeads was taken into culture on 0.1% gelatin coated material with SP^++^ medium [EBM-2 (Lonza, CC-3162) mixed 1:3 with M199 (Gibco, #31150-030)] supplemented with 10% fetal calf serum and 1% non-essential amino acids (Gibco, # 11140-035), complemented with 10 ng/ml basic fibroblast growth factor (Sigma-Aldrich, F0291).

CMPCs were cultured at 37°C and 5% CO_2_ in non-differentiating conditions and passed twice a week without bFGF, in non-differentiating conditions when confluency reached 50-80%, without losing their differentiating abilities. These cells have previously been characterized by Goumans et al. (2007). CMPCs were dissociated with trypsin (Gibco #25200-056) prior to being seeded on MEAs or coverslips. CMPCs used for experiments were in passage 10–15. Conditioned medium (Cme) was obtained by culturing CMPCs in 2% FBS culture medium for 24 h. This medium was filtered (0.22 μm), and stored at −20°C if not used directly.

### (Co)culture conditions

7.0 × 10^3^ CMPCs were seeded (in a fixed and predetermined manner) onto MEAs in a circular pattern with a surface area of 2.46 cm^2^ reaching a 50–70% confluency (Supplemental Figure 1). The next day, freshly isolated NRVMs, as described above, were seeded onto the MEAs. NRVMs only functioned as control monolayers, whereas NRVMs seeded over the cluster of CMPCs, formed the co-culture (NRVM + CMPC) condition. The subsequent day (co)cultures were washed to remove dead cells. Five days after seeding NRVMs, cultures were used for electrical mapping and micro-electrode studies. Conditioned medium of CMPCs was added to control monolayers 48 h prior to electrical mapping and micro-electrode studies, these cultures were referred to as Cme CMPC.

### Electrical mapping and microelectrode measurements

Electrophysiological parameters were determined by mapping the spontaneous electrical activity of the (co)cultures. Unipolar electrograms were recorded with a 256-channel amplifier (BioSemi, ActiveTwo, Amsterdam, The Netherlands, 24-bit dynamic range, 122.07 nV LSB, total noise 0.5 μV). Signals were recorded with a sampling frequency of 2,048 Hz (filter setting of the amplifiers DC – 400 Hz [−3 dB point]). The recordings were made with respect to an external reference electrode placed inside the MEA.

At each local electrogram activation time (AT) was determined at the time of the minimum derivative of the local RS, using a custom made program (Potse et al., 2002) based on MATLAB R2006b (The MathWorks, Inc., Natick, MA, USA). The minimum derivative (dV/dt) threshold was set at −0.1 mV/ms. Recordings that did not meet this threshold or contained too much noise were excluded from analysis. Activation maps were constructed using the AT, conduction velocity (CV) was determined along lines perpendicular to isochronal lines by dividing the distance by the difference in local AT. Lines had a minimum length of 4 electrode distances. Local activation times in the figures are color coded in classes of 10 ms. Isochronal lines were drawn by hand at 15 ms intervals.

We took the electrode with the earliest AT to represent the origin of spontaneous activity. The electrode demonstrating the earliest AT was recorded for each individual MEA to construct a map illustrating the origin of spontaneous activity in the different cultures. Cultures demonstrating a high rate of spontaneous activity (cycle length <450 ms) were classified as having tachy-arrhythmias.

Electrograms, recorded with the MEA-system, demonstrating an elevated ST-segment or demonstrating monophasic action potential-like signals were classified as having ST-elevation (Supplemental Figure 2). The ST potential was determined from the local electrograms relative to a zero potential at the mid-diastolic segment. For each electrogram, it was examined whether the ST-segment was at 0.1 mV, below or above 0.1 mV amplitude. Per MEA the electrodes with ST-elevation (ST > 0.1 mV) were documented. Per electrode, the percentage of MEA, showing ST-elevation on this same electrode was calculated, resulting in a map indicating what the incidence (%) of ST-elevation is per electrode.

Glass pipette microelectrodes (Harvard apparatus GC100F-10) were used to record APs. These micro-electrodes were pulled from glass capillaries and filled with 3 M KCl. Impedance was between 15 and 25 MΩ. An AgCl covered silver wire was used as a reference electrode. RMP was defined as the most negative diastolic membrane potential recorded. Upstroke velocity was defined as maximum dV/dt and action potential duration was determined at 50% (APD50) of the AP.

### Immunostainings

For immunostainings, a mixed population of CMPCs and NRVMs was made. Briefly, 7.0 × 10^3^ CMPCs were seeded onto fibronectin coated coverslips. The next day, 1.5 × 10^5^ NRVM were added and co-cultures were cultured the same as the MEA co-cultures. Cultures were fixed in 4% PFA, before permeabilizing (0.1% Triton) and blocking (1% horse serum/PBS) steps were performed. Cultures were stained with primary antibodies [rabbit anti Connexin 43 (Invitrogen 574366A; 1:200), goat anti cTnT (Hytest LD 16/06-4T19/2; 1:1000X), and mouse anti-beta integrin (Santa Cruz, SC-53711; 1:1,000)] overnight at 4°C before incubation with secondary antibodies (Alexa Fluor-647 donkey anti-mouse IgG (Life Technologies, A31571; 1:250), Alexa Fluor-488 donkey anti-rabbit (Life Technologies, A21206, 1:250) and Alexa Fluor-568 donkey anti goat IgG (Life Technologies, A11057; 1:250), for 2 h in 1% horse serum/PBS. Nuclei were stained with DAPI (Sigma-Aldrich, D9542, 1:40,000). Examination was performed using Leica SPE confocal laser scanning and Leica Application Suite Advanced Fluorescence software.

### Statistical analysis

The data (CV, upstroke velocity, and APD50) had a continuous and normal distribution in the control group and Cme CMPC group, yet a skewed distribution for the NRVM + CMPC group and thus data are presented as mean ± SEM but tested with a Kruskal-Wallis analysis using the Dunn's test as the *post-hoc* analysis. Nominal values expressed as percentages were compared with a χ^2^ tests or the Fisher-Exact test for comparisons when expected cell frequency was < 5. A *p* < 0.05 was considered statistically significant. Graphs were made using GraphPad Prism software version 5 (Graphpad Software, La Jolla, CA, USA).

## Results

### NRVM+CMPC co-cultures are depolarized

**Figure 1:**
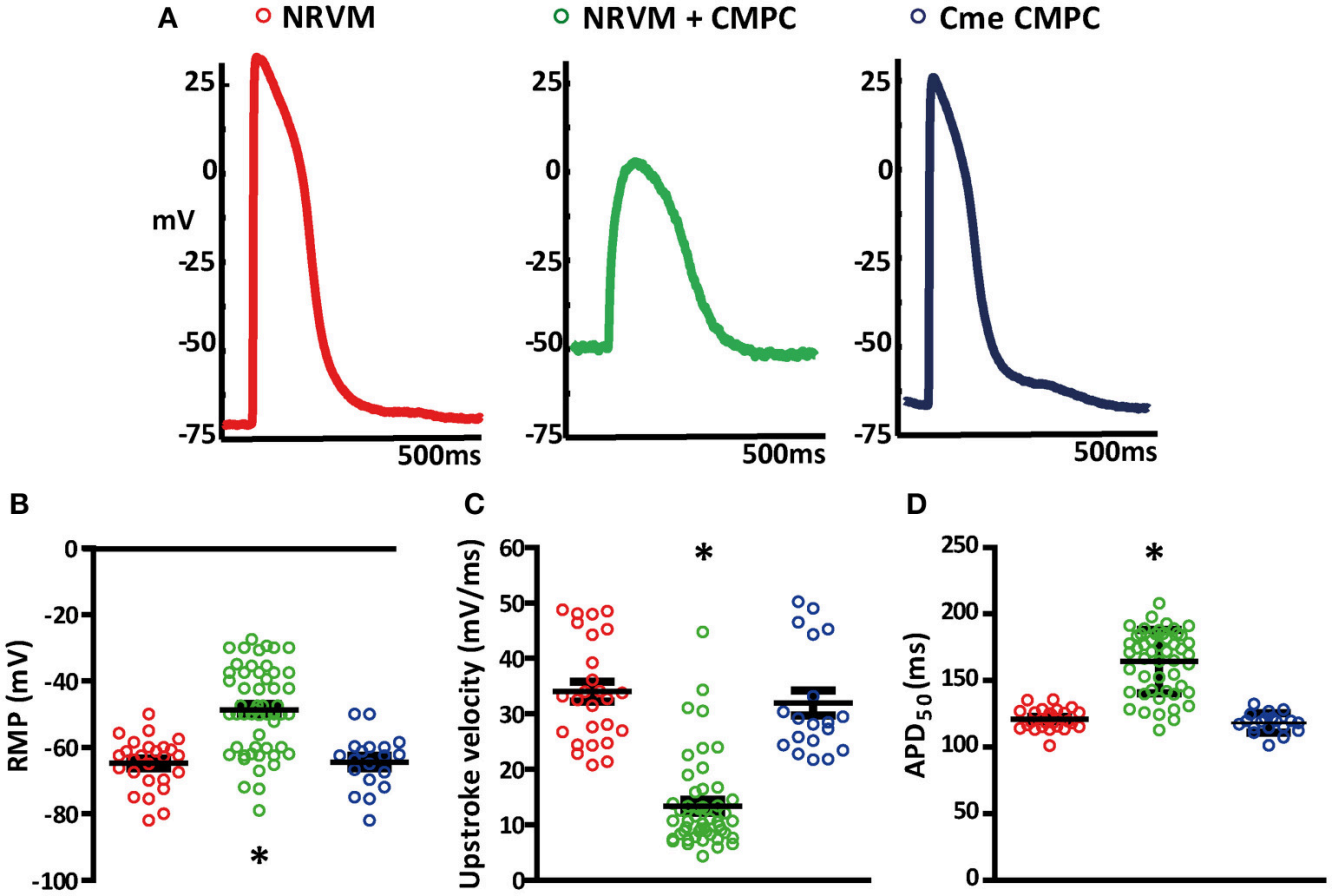
Effects of CMPCs or Cme on the action potential. **(A)** Typical examples of action potentials recorded in control monolayers (red tracing), co-cultures (green tracing) and monolayers cultured in Cme (blue tracing). Scatter-plots illustrating the effect of CMPCs and Cme on **(B)** resting membrane potential, **(C)** upstroke velocity, and **(D)** APD50. APD50, action potential duration at 50% of the action potential; Cme, conditioned medium; CMPC, cardiomyocyte progenitor cells; NRVMs, neonatal rat ventricular myocytes; and RMP, resting membrane potential. The ^*^indicates a *p* < 0.001 compared to both control monolayers and monolayers cultured in Cme CMPCs.

Figure 1A shows an example of APs recorded during micro-electrode measurements in each of the conditions investigated. APs recorded in control monolayers and monolayers of NRVMs cultured in Cme CMPC demonstrated ventricular-type APs (Figure 1A, left and right panel). The APs recorded from the area overlying the CMPCs in the co-cultures demonstrated a more depolarized AP, with a slower upstroke velocity and a smaller amplitude (Figure 1A, middle panel).

Overall, APs recorded from the co-cultures (*n* = 53 APs) had less negative RMP than control NRVM monolayers (*n* = 26, −47.3 ± 1.8 vs. −64.8 ± 1.5 mV, *p* < 0.001, Figure 1B). RMP in monolayers of NRVMs cultured in Cme CMPC (*n* = 19) was on average −64.4 ± 1.9 mV and differed significantly from the co-cultures (*p* < 0.001, Figure 1B) but not from controls (*p* = 1.00, Figure 1B). RMP was normally distributed in all three group (Shapiro-Wilks test: NRVM: 0.50, NRVM+CMPC: 0.08 and Cme CMPC: 0.57). Upstroke velocity was calculated from the recorded APs. On average, the upstroke velocity in control NRVM monolayers was 34.0 ± 1.8 mV/ms and 31.9 ± 2.3 mV/ms in monolayers of NRVMs cultured in Cme CMPC (*p* = 0.346, Figure 1C). Co-cultures demonstrated significantly reduced upstroke velocities (13.3 ± 1.1 mV/ms) compared to control NRVM cultures (*p* < 0.001, Figure 1C) and monolayers cultured in Cme CMPC (*p* < 0.001, Figure 1C). Furthermore, co-cultures demonstrated prolonged APD50 compared to control NRVM monolayers (164.3 ± 3.3 vs. 120.9 ± 1.5 ms, *p* < 0.001 Figure 1D) and monolayers cultured in Cme CMPC (118.2 ± 1.7 ms, *p* < 0.001, Figure 1D).

### Co-cultures demonstrate conduction slowing

**Figure 2:**
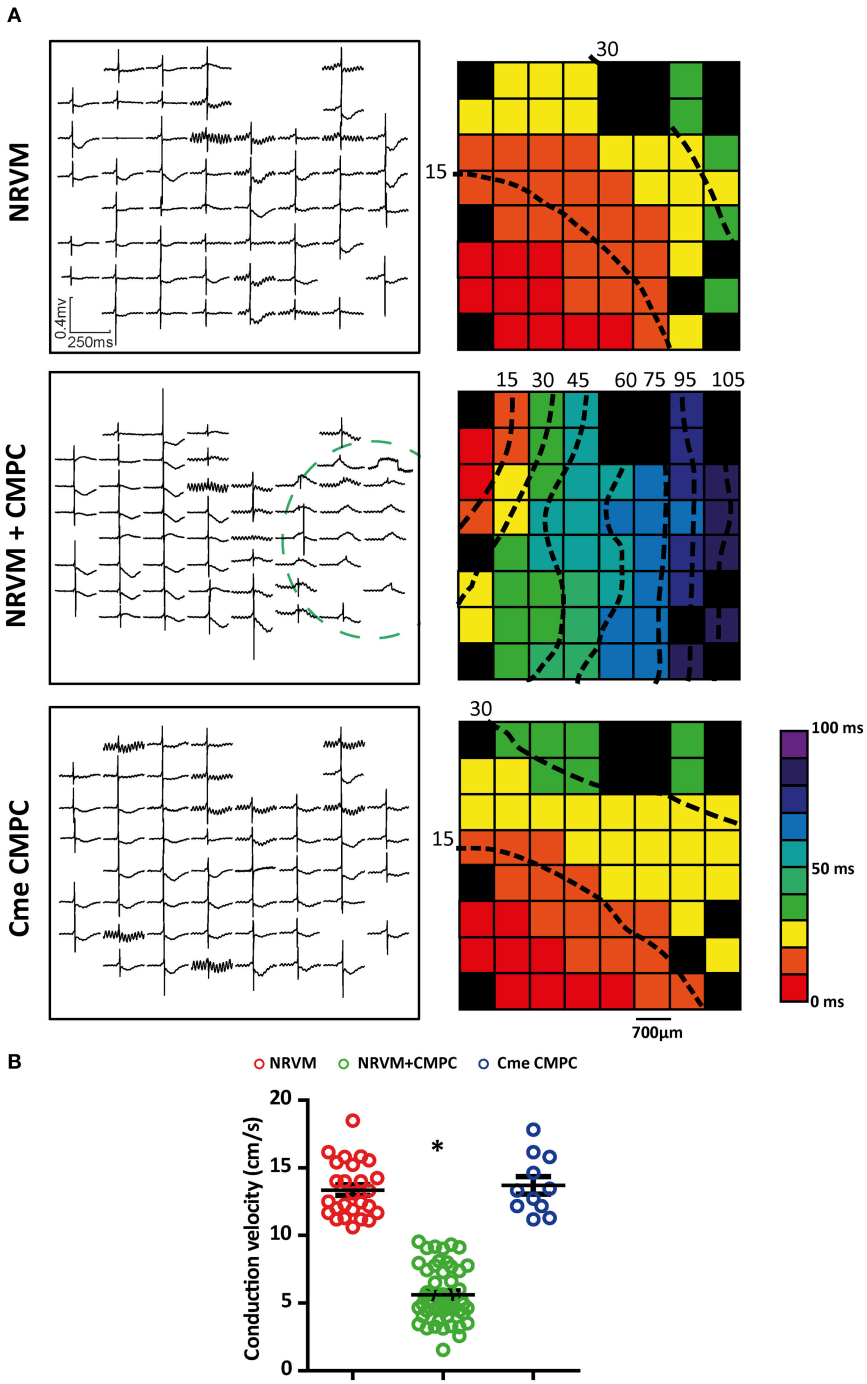
Effects of CMPCs or Cme on conduction velocity. **(A)** An example of the local electrograms recorded and the corresponding activation map for each of the different cultures investigated. **(B)** A scatter-plot showing the effects of CMPCs and Cme CMPC on conduction velocity. Conduction velocity is calculated along lines perpendicular to the isochronal lines. Black boxes indicate electrodes excluded from analysis and black dashed lines represent isochrones lines. Cme, conditioned medium; CMPCs, cardiomyocyte progenitor cells; and NRVMs, neonatal rat ventricular myocytes. The ^*^indicates a *p* < 0.001 compared to both control monolayers and monolayers cultured in Cme CMPC.

We next studied whether the depolarization resulted in conduction slowing. Examples of local electrograms recorded in the different conditions investigated are shown in Figure 2A. The dotted circle in the middle panel denotes the region where CMPCs were seeded. Note that the electrogram morphology is homogeneous with a short QRS-complex in both the control monolayers and those subjected to Cme (top and bottom panel, respectively). The middle panel shows that the local electrograms from the co-cultured region demonstrate delayed activation and local ST-elevation. Activation maps were created and CV was calculated. Overall, monolayers of NRVMs co-cultured with CMPCs demonstrated spatially heterogeneous conduction slowing compared to control monolayers of NRVMs (Figure 2A middle panel and top panel, respectively) or monolayers subjected to Cme (bottom panel). On average, CV was 13.4 ± 0.4 cm/s in monolayer of NRVMs (*n* = 26 monolayers), and 5.6 ± 0.3 cm/s in co-cultures (*n* = 50 monolayers, *p* < 0.001, Figure 2B). Monolayers cultured in Cme CMPC were not different from control monolayers (*n* = 11 monolayers, 13.7 ± 0.6 cm/s, *p* = 1.00, Figure 2B). CV in co-cultures was significantly lower compared to monolayers cultured in Cme CMPC (*p* < 0.001, Figure 2B).

### Co-cultures demonstrate ST-elevated electrograms in the CMPC region

**Figure 3:**
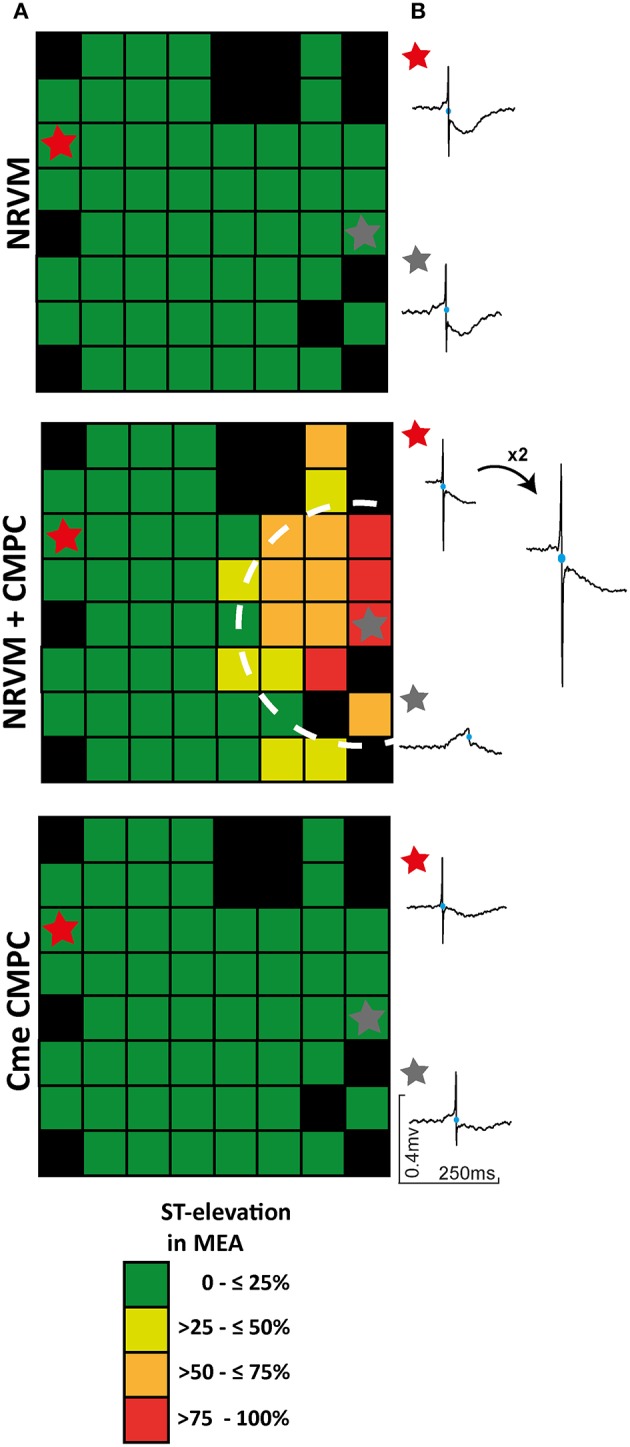
The effect of CMPCs on ST-elevation in local electrograms. **(A)** ST-elevation maps illustrating the percentage of MEAs that displayed ST-elevation per electrode. **(B)** An example of two local electrograms recorded in either a control monolayer, a co-culture or a monolayer of NRVMs cultured in Cme CMPC, respectively. Black boxes indicate electrodes excluded from analysis and blue circles on the local electrograms indicate the earliest activation time. In one example the RS-complex was enlarged to show the negative flank of the RS complex. Cme, conditioned medium; CMPCs, cardiomyocyte progenitor cells; MEA, multi-electrode array; and NRVMs, neonatal rat ventricular myocytes.

In general, local electrograms from NRVM monolayers display a negative ST-segment transiting immediately into a negative T-wave (Figure 2A). Local electrograms with ST-elevation were apparent in the CMPC area of co-cultures (Figures 2A, 3, circled area) but not in control monolayers or monolayers of NRVMs cultured in Cme CMPC. Because a quantitative analysis of the potential gradients in the ST-segment in these cultures is not possible (because of the gradual sloping into the T-wave) we constructed qualitative pooled ST-elevation maps, maps illustrating the percentage of MEAs demonstrating ST-elevation on a specific electrode (each square represents *n* = 11–50 electrograms, Figure 3A). In control monolayers (*n* = 26) and monolayers cultured in Cme CMPC (*n* = 11) ST-elevations are typically not seen, and if present they do not occur in a regional pattern (Figure 3A). However, co-cultures (*n* = 50) demonstrated electrograms with ST-elevation specifically in the region where CMPCs were seeded, whereas local electrograms in the unmodified region show normal ST segments (Figure 3B).

### Origin of earliest activation and arrhythmias

**Figure 4:**
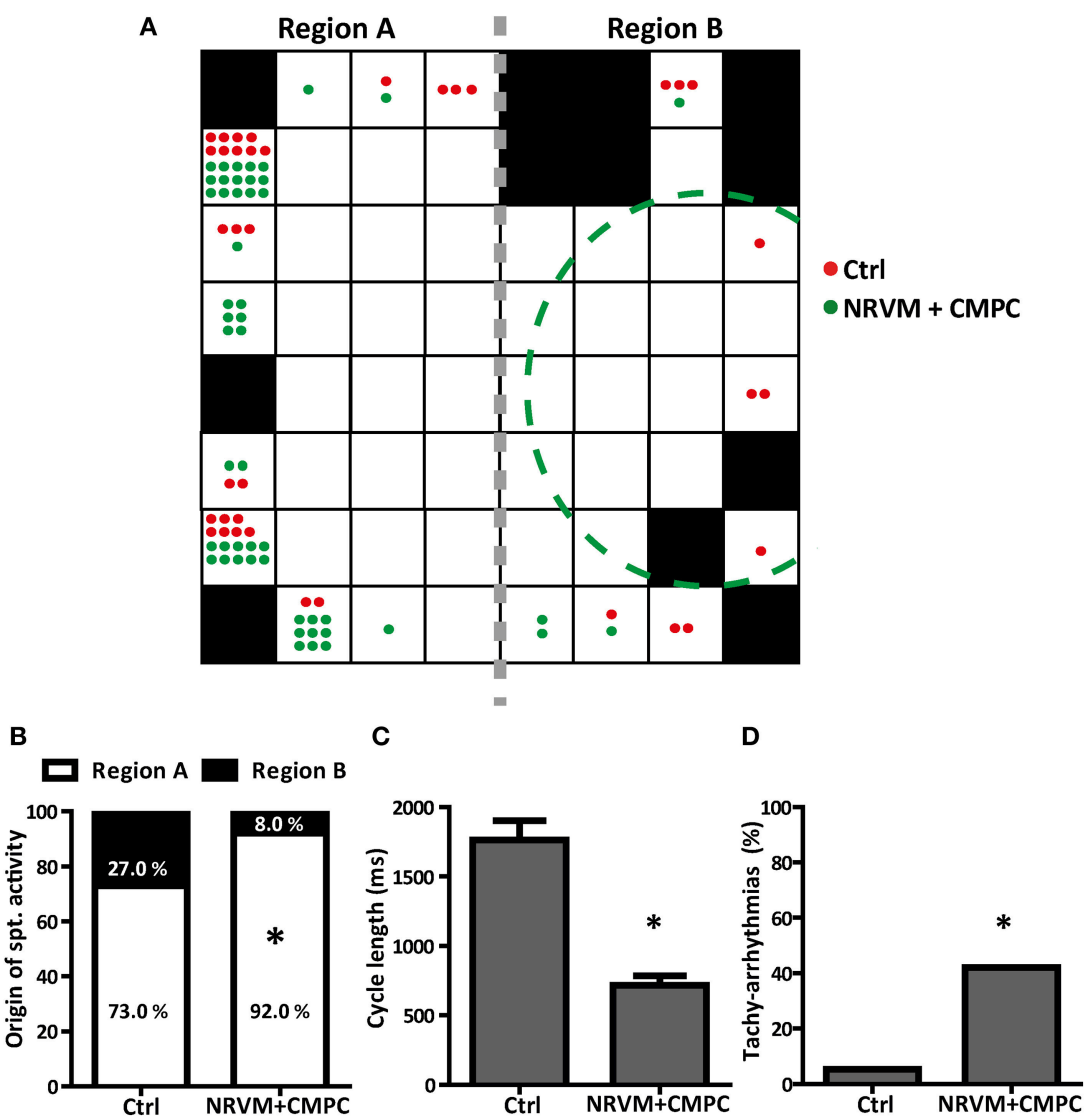
The effects of CMPCs on the origin of spontaneous activation, the cycle length and the occurrence of tachy-arrhythmias. **(A)** A MEA illustrating the electrodes with the origin of the earliest AT per MEA studied for control cultures (red dots) and co-cultures (green dots). The gray dashed line indicates where the MEA is split into region A and region B. Black boxes indicate electrodes excluded from analysis. **(B)** Percentage of MEAs with earliest AT in region A and region B. **(C)** Average cycle length of the spontaneous rhythm and **(D)** The percentage of MEAs that displayed tachy-arrhythmias (cycle length <450 ms). AT, activation time; Cme, conditioned medium; CMPCs, cardiomyocyte progenitor cells; MEA, multi-electrode array; and NRVMs, neonatal rat ventricular myocytes. The ^*^indicates a *p* < 0.001 compared to control monolayers.

Cultures of NRVMs usually beat spontaneously (Rohr et al., 1991). We next assessed the influence of CMPCs on spontaneous activity, reasoning that a diastolic “injury”-like current flowing intracellularly from the depolarized to normal region, would accelerate spontaneous activity in the unmodified region (Janse et al., 1980). In all conditions, spontaneous activity originated from one of the outer electrodes and never from the center of the preparation (Figure 4A). The MEA electrodes were grouped into two regions; Region A consisting of NRVMs only and Region B consisting of NRVMs co-cultured with/without CMPCs (Figure 4A; see the gray dashed line separating the two regions). Because monolayers of NRVMs cultured in Cme CMPC demonstrated similar CV, RMP, upstroke velocities and APD50, we pooled this group with the NRVM monolayers for activation mapping analysis, to create one control group (*n* = 37 monolayers). In these control cultures, the origin of spontaneous activity was not significantly different between region A and B. In co-cultures (*n* = 50), spontaneous activity originated more often from region A than from region B (92.0 vs. 8.0%, in comparison to the distribution in controls 73.0 vs. 27.0%, *p* < 0.01, Figure 4B).

The average cycle length of the spontaneous rhythm was 1,761.9 ± 140.9 ms in control (*n* = 37) and 716.8 ± 69.0 ms in co-cultures (*n* = 50, *p* < 0.001, Figure 4C). The percentage of spontaneous rhythm with a cycle length <450 ms (tachy-arrhythmias) was calculated for all MEAs that were spontaneously active. Co-cultures demonstrated a significantly higher occurrence of tachy-arrhythmias than control monolayers, (42.0% vs. 5.4%, *p* < 0.001, Figure 4D).

### Cell characterization and connexin 43

**Figure 5:**
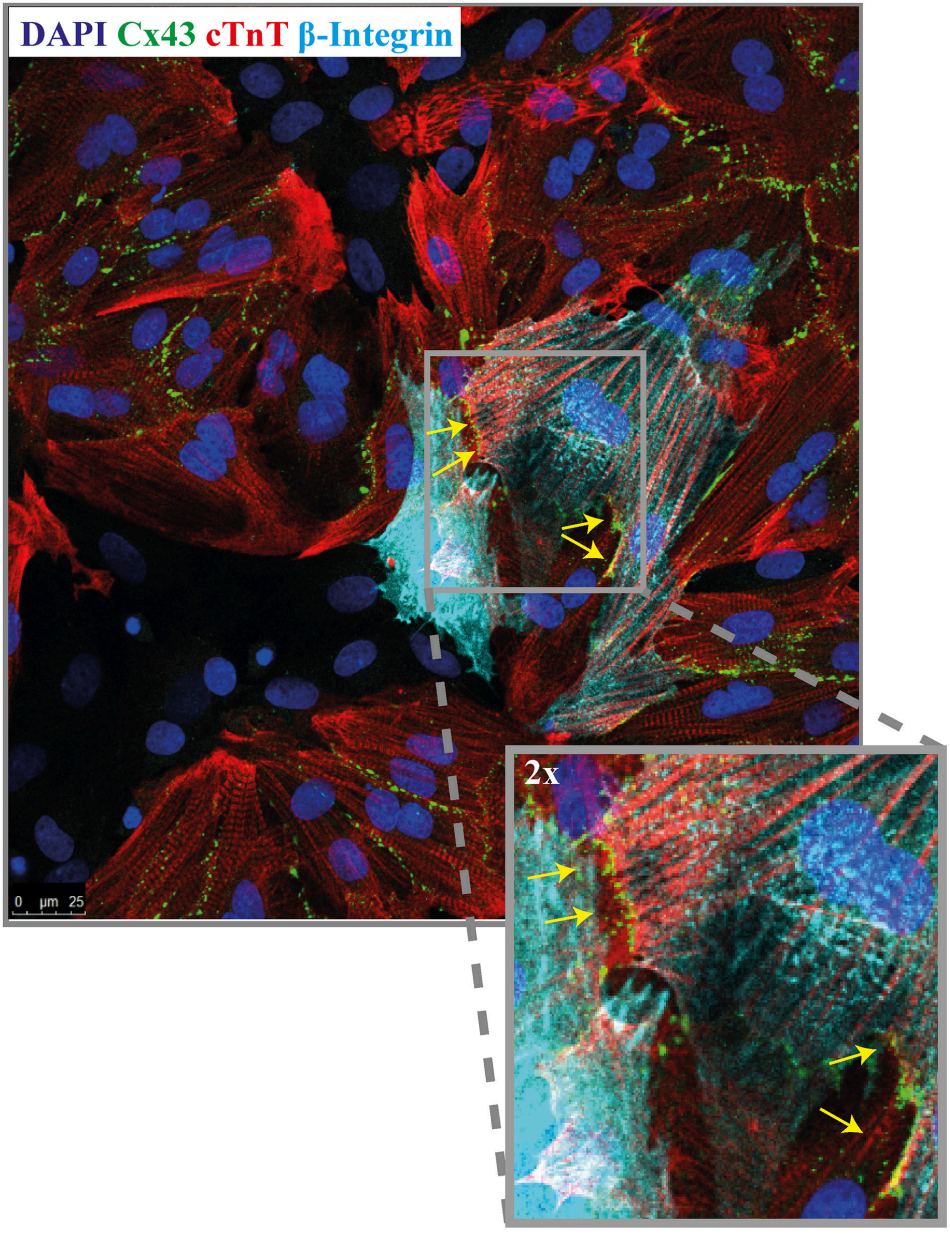
Immunofluorescence micrograph of a co-culture stained for Cx43. Co-cultures are stained for nuclei (dapi, blue), NRVMs (cTNT, red), CMPCs (human beta-integrin, light blue) and Cx43 (green). The yellow arrows indicates the presence of Cx43 between a NRVM and a CMPC. (Orginal magnification, 40x). CMPCs, cardiomyocyte progenitor cells; cTnT, cardiac troponin; DAPI, 49.6-diamidino-2-phenylindoleh; and NRVMs, neonatal rat ventricular myocytes.

Immunofluorescence staining was performed to demonstrate the presence of the gap junctional protein connexin43 (Cx43). For this purpose, co-cultures of NRVMs and CMCPs were made with lower seeding densities and dispersed cells so that the connexins between cells could be visualized. NRVMs were distinguished by cTnT staining and CMPCs by human specific beta-integrin staining. Figure 5 illustrates Cx43 presence between NRVMs and CMPCs (Figure 5, indicated with the yellow arrows).

## Discussion

In this study, we demonstrated that undifferentiated, human cardiomyocyte progenitor cells form a pro-arrhythmic substrate when cultured together with neonatal rat ventricular myocytes. Micro-electrode measurements revealed that CMPCs created a depolarized region that was characterized by APs with severely reduced upstroke velocities and prolonged APD. Our data indicate that CMPCs are coupled to NRVMs. Due to coupling, a depolarized region formed that led to a diastolic flow of an “injury”-like current toward the normal tissue, evident from the observations of (1) the presence of conduction slowing (2) ST-segment changes recorded in the area with regional depolarization, (3) a higher incidence of tachy-arrhythmias and, supported by the detection of Cx43 between NRVMs and CMPCs. Cme obtained from the same CMPCs did not reproduce any of these findings strengthening the conclusion that the CMPCs form a pro-arrhythmic substrate through coupling with cardiomyocytes. The arrhythmogenic substrate is caused by two different arrhythmogenic mechanisms. First, regional conduction slowing makes the myocardium more susceptible to reentrant arrhythmias. Second, the flow of “injury”-like current will accelerate spontaneous rhythms by providing an additional depolarizing current to the non-depolarized tissue. Both mechanisms, alone and in combination, can lead to lethal ventricular arrhythmias (Janse et al., 1980).

Micro-electrode recordings demonstrated that the cultures consisting of NRVMs and CMPCs were depolarized compared to control monolayers (Figure 1B). The relatively large spread in RMP in the co-culture condition can be explained by the fact that micro-electrode measurements were done throughout the entire MEA. This includes the region dominated by NRVMs as well as the region that consisted of both the CMPCs and NRVMs. Importantly, we did not record a bimodal distribution of RMP. A bimodal distribution was to be expected if coupling did not occur between the CMPCs and the NRVMs. Our observations indicated that the RMPs have a normal distribution and that the two cell types, therefore, were effectively coupled. The heterogeneity in cell types is further strengthened by the large variability of the morphology of the AP recorded in the co-cultures.

The RMP is important because it impacts on the availability of the voltage-dependent sodium channels for activation. Coupling of cardiomyocytes to depolarized inexcitable cells with low RMP is expected to cause electrotonic current from a cell with a less negative RMP (CMPC) to one with a more negative RMP (NRVM), resulting in a less negative RMP of the cardiomyocyte, reduction in the upstroke velocities of its AP, and APD prolongation. These changes were indeed seen in our co-cultures (Figure 1). When CMPCs induce severe membrane depolarization, as is seen in the CMPC cluster region, this can cause (local) in-excitability, resulting in a monophasic-like local electrograms (Franz, 1999; Coronel et al., 2006) and a reduced CV. This phenomenon is supported by activation mapping.

The local cluster of CMPCs formed a depolarized region compared to the purely NRVM region in the same MEA. The difference in RMP between the two regions leads to an intracellular current flow, the “injury”-like current (Samson and Scher, 1960). Because of the diastolic intercellular current flow, there is the opposite change in voltage in the extracellular space, which is detected and recorded by our electrical mapping system as TQ-depression (Vincent et al., 1977). Because the signals were necessarily set to zero in diastole, these changes are reflected as ST-elevation in the depolarized region and ST-depression in the normal area (Coronel et al., 1991). The diastolic current of “injury” is expected to speed the spontaneous depolarization rate in the non-depolarized tissue. Consistent with the “injury”-like current, we observed ST-elevations specifically in the region where the CMPCs were seeded and not in regions that contained only NRVMs (in the same MEA, Figure 3).

Together with ST-elevations, we expected to also observe electrograms with ST-depressions, specifically near the border zone between the depolarized region and the NRVM region. This observation was not as prominently present as the ST-elevations. We reasoned that this is due to the geometry of the depolarized region (a small circle in a larger monolayer). The current flowing between the regions is thus more dissipated in the normal myocyte region than in the depolarized region. This explains why electrograms with ST-elevations are more observable than the ST-depressions. An important strength of our approach is that the ST-elevation phenomenon was only observed because we used electrical mapping techniques which allow us to record the changes in the extracellular space. Optical mapping with fluorescent, voltage sensitive dyes does not permit these analyses.

The diastolic “injury”-like current is also known to increase the rate of spontaneous activity in the (normal) region next to the depolarized region (Janse and van Capelle, 1982). We did indeed observe a faster rate of spontaneous activity in co-cultures but not in control preparations. Secondly, the co-cultures had a higher incidence of tachy-arrhythmias compared to controls. Third, we have shown that the earliest activation, in co-cultures, predominantly occurs in region A, the region dominated by the NRVMs, and not in Region B, the depolarized region (Figure 4A). This suggests that the tachy-arrhythmias originate from the NRVM region, albeit on the sides of the MEA. We assume that the earliest AT is always on the “outer” marginal region of the MEA as at these places the cells have less neighboring cells making it easier to activate as there is less current to load mismatch. This current to load mismatch is more like to occur away from the outer sides as there would be more neighboring cells that need to be activated (Rohr, 2004). The effect of the “injury”-like current, although present in the entire preparation, become only evident at sites with the fastest rate of diastolic depolarization, where it will result in a faster spontaneous rate of diastolic rate of depolarization and a faster beating rate. In our preparations, these sites were located at the margin of the cultures.

Secondly, it may be argued that rodent cardiomyocytes beat faster than human cardiomyocytes (Milani-Nejad and Janssen, 2014) explaining why we see arrhythmia-like electrophysiology in our cultures. However, neonatal rat cells beat less rapid than their adult form and they are shown to be a suitable model for electrophysiological studies (Rohr et al., 1991; Fast and Kleber, 1994). This is can also be seen from the cycle length data, where our control cultures have an average cycle length of 1,761.9 ± 140.9 ms.

Cme of the CMPCs did not alter the RMP, upstroke velocity, APD50, CV, or the ST-segments in the electrograms recorded and values did not differ from controls. This is in line with our previous study in which Cme obtained from human adipose tissue-derived stromal cells did not elicit heterogeneous conduction slowing, which was seen when the cells were used (Ten Sande et al., 2016). It supports the notion that the present observed effects are caused by coupling between the NRVMs and the CMPCs and not by paracrine effects. However, paracrine communication between cells is extremely complex and it is certainly possible that pathways initiated by paracrine factors can be influenced by, e.g., different cellular adhesions. Secondly, the secretion of paracrine factors can also be influenced by the paracrine or autocrine response of different cells in the environment (Smit et al., 2017). We argue that by using media conditioned by CMPCs we demonstrate that direct paracrine release, without cellular adhesion to different cells, is not pro-arrhythmic, but does not *per se* exclude any paracrine signaling depending on the level of adhesion in co-cultures.

The immuno-fluorescence data demonstrate the presence of Cx43 between CMPCs and NRVMs. Undifferentiated CMPCs are known to express Cx43 (Goumans et al., 2007). The presence of Cx43 but foremost the presence of the ST-elevations in the electrograms overlying the CMPCs, together with the depolarized RMP and conduction slowing provide strong evidence that functional coupling between the CMPCs and NRVMs occurred.

## Conclusions

In the current study, we have shown that locally seeded, undifferentiated, human CMPCs form a pro-arrhythmic substrate when co-cultured with cardiomyocytes. The progenitor cells used are known to express Cx43 allowing them to couple with cardiomyocytes. Coupling with cardiomyocytes results in the flow of a diastolic “injury”-like current driven by membrane potential differences between both regions. Our study demonstrates that CMPCs can contribute to the formation of a pro-arrhythmic substrate through two different mechanisms (regional conduction slowing and speeding of spontaneous rhythms). Therefore, the use of CMPCs should be made with precaution. The conditioned medium did not induce any of the above effects.

## Author contributions

NS: experimental design, data acquisition, analysis and interpretation, drafting and editing manuscript, and approval. LC: experimental design, data acquisition, analysis and interpretation, editing manuscript, and approval. AV and AS: data acquisition and interpretation, editing manuscript, and approval. VM: analysis and interpretation of data, editing manuscript, and approval. MK: experimental design, data acquisition, editing manuscript, and approval. AT, HT, MG, and GB: experimental design, data interpretation, editing manuscript, and approval. RC: experimental design, data analysis and interpretation, editing manuscript, and approval.

### Conflict of interest statement

The authors declare that the research was conducted in the absence of any commercial or financial relationships that could be construed as a potential conflict of interest.

## Abbreviations

AP: action potential
APD: action potential duration
APD50: action potential duration at 50% of the action potential
AT: activation time
Cme: conditioned medium
CMPCs: cardiomyocyte progenitor cells
CSCs: cardiac stem cells
cTnT: cardiac troponin
CV: conduction velocity
Cx43: connexin 43
DAPI: 49.6-diamidino-2-phenylindoleh
MEA: multielectrode array
mV: milliVolts
NRVMs: neonatal rat ventricular myocytes
RMP: resting membrane potential.
