# Supplementary material for: Human Cardiomyocyte Progenitor Cells in Co-culture with Rat Cardiomyocytes Form a Pro-arrhythmic Substrate: Evidence for Two Different Arrhythmogenic Mechanisms
Source: Front Physiol. 2017 Oct 12;8:797. doi: 10.3389/fphys.2017.00797 (PMC5644204; doi:10.3389/fphys.2017.00797)
Supplement: Supplementary file 1 [file Presentation1.pdf]

Supplementary Material

Human Cardiomyocyte Progenitor Cells in Co-Culture with Rat Cardiomyocytes Form a Pro-arrhythmic Substrate: Evidence For Two Different Arrhythmogenic Mechanisms.

Nicoline W. Smit, Lucia Cócera Ortega, Anna M.D. Végh, Veronique M.F. Meijborg, Anke M. Smits, Mischa Klerk, Anke J. Tijsen, Hanno L. Tan, Marie-José H.T. Goumans, Gerard J.J. Boink, Ruben Coronel*

*** Correspondence:** Ruben Coronel: rubencoronel@gmail.com

1 Supplementary Figures and Tables

1.1 Supplementary Figures

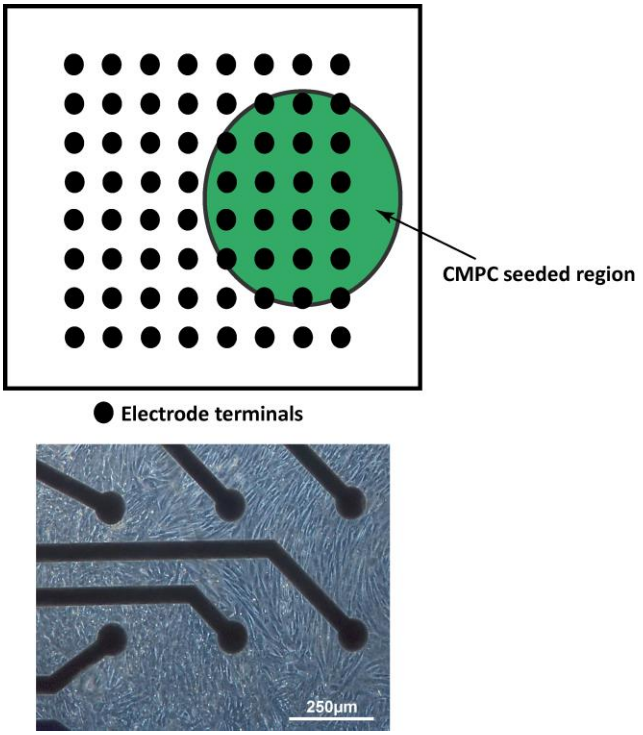

Supplemental Figure 1. Schematic representation of the CMPC location. Black dots represent electrodes in the MEA. Each electrode had a diameter of 100 µm and an inter-electrode distance of 700µm. The green circle indicates the standard position where the cluster of CMPCs were seeded. A light microscope image of a co-culture of NRVM and CMPCs, original magnification is 10x. Abbreviations: CMPCs: cardiomyocyte progenitor cells, and MEA: multi-electrode array.

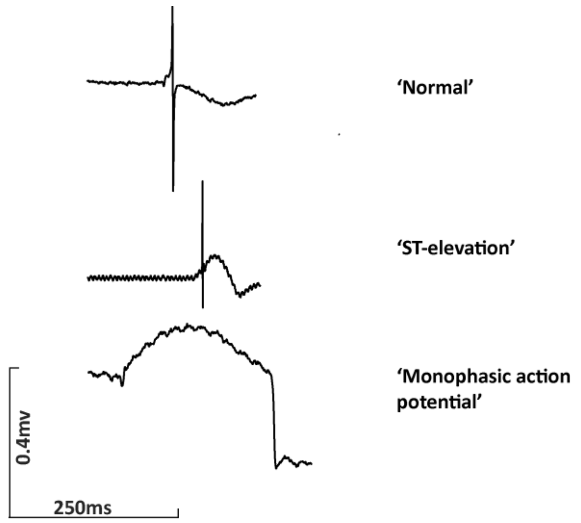

Supplemental Figure 2. Electrograms showing ST-elevation or electrograms with monophasic action potentials. An example of a normal electrogram, an electrogram with ST-elevation and an electrogram demonstrating a 'monophasic action potential'.
